# Quantitative Proteomics of *Spodoptera frugiperda* Cells during Growth and Baculovirus Infection

**DOI:** 10.1371/journal.pone.0026444

**Published:** 2011-10-18

**Authors:** Nuno Carinhas, Aaron Mark Robitaille, Suzette Moes, Manuel José Teixeira Carrondo, Paul Jenoe, Rui Oliveira, Paula Marques Alves

**Affiliations:** 1 Instituto de Tecnologia Química e Biológica-Universidade Nova de Lisboa/Instituto de Biologia Experimental e Tecnológica, Oeiras, Portugal; 2 Department of Biochemistry, Biozentrum of the University of Basel, Basel, Switzerland; 3 Departamento de Química, Faculdade de Ciências e Tecnologia, Universidade Nova de Lisboa, Caparica, Portugal; 4 REQUIMTE, Systems Biology and Engineering Group (SBE), Departamento de Química, Faculdade de Ciências e Tecnologia, Universidade Nova de Lisboa, Caparica, Portugal; University of Cambridge, United Kingdom

## Abstract

Baculovirus infection *of Spodoptera frugiperda* cells is a system of choice to produce a range of recombinant proteins, vaccines and, potentially, gene therapy vectors. While baculovirus genomes are well characterized, the genome of *S. frugiperda* is not sequenced and the virus-host molecular interplay is sparsely known. Herein, we describe the application of stable isotope labeling by amino acids in cell culture (SILAC) to obtain the first comparative proteome quantitation of *S. frugiperda* cells during growth and early baculovirus infection. The proteome coverage was maximized by compiling a search database with protein annotations from insect species. Of interest were differentially proteins related to energy metabolism, endoplasmic reticulum and oxidative stress, yet not investigated in the scope of baculovirus infection. Further, the reduced expression of key viral-encoded proteins early in the infection cycle is suggested to be related with decreased viral replication at high cell density culture. These findings have implications for virological research and improvement of baculovirus-based bioprocesses.

## Introduction

Baculoviruses (BVs) are a diverse family of arthropod pathogens containing large DNA genomes reaching up to 180 kbp [1]. BVs vary considerably in their genome composition and in general have a narrow host range; the prototype *Autographa californica* multicapsid nucleopolyhedrovirus (*Ac*MNPV) infects a few insect lepidopteran species. The economic interest of BVs derives from their use as biopesticides and, along the past decades, as versatile protein expression systems [2]. Due to their inability to replicate in mammalian cells, their use as antigen displaying vectors and gene delivery vehicles has become exceedingly popular in recent years [3]. The host for *AcMNPV* propagation is usually a cell line derived from the fall armyworm *S. frugiperda*, particularly the *Sf*9 clonal isolate. More than simply a vehicle for viral propagation, insect cell lines are chosen for their robust growth, straightforward culture scale-up and post-translational modifications of expressed proteins [4].

Knowledge on the BV infection cycle has accumulated over many years of research. Briefly, replication and assembly take place in the nucleus, where viral particles travel by reorganizing the actin cytoskeleton upon cell entry [5]. The molecular life cycle is broadly divided in three consecutive phases according to gene expression programming. Early genes (0–6 hours post-infection (hpi)), requiring the transcriptional activity of the cell-encoded RNA polymerase II, mainly act as master transactivators essential for both subsequent viral gene expression and subversion of host cell activity by performing tasks common to other DNA viruses, including cell cycle arrest [6], [7]. Block of apoptosis by the viral protein P35 [8] is also a required activity to establish productive infections. The transition from early to late phase is marked by the onset of viral DNA replication (6–18 hpi) and the activity of a virus-encoded, α–amanitin resistant RNA polymerase [9]. Concomitant with the onset of the very late phase (18 hpi–), a pronounced down–regulation of host transcriptional activity occurs [10], followed by a shut off of most protein synthesis by 24 hpi [11]. This phenomenon reflects increased viral autonomy during the later phases of infection, confirmed by the observation that translation of the very-late genes *p10* and *polh* is relatively insensitive to the presence of 5′-cap-binding eukaryotic initiation factor eIF4E [12], [13].

In spite of the biotechnological interest, the genomes of *Ac*MNPV susceptible cell lines have not been sequenced. This constitutes a major disadvantage for the use of genome–wide technologies for better understanding the host-pathogen interplay. So far, global expression patterns of *Sf*9 cells at mRNA level have been recorded at various time-points following infection [14], [15]. Also, a recent proteomic analysis based on 2D–gel electrophoresis (2DGE) identified 18 (out of 21) proteins differentially expressed 24 h after *Ac*MNPV infection in a different, permissive cell line [16].

Among the “omic” technologies available, quantitative proteomics based on stable isotope labeling by amino acids in cell culture (SILAC) has become a preferred choice for large–scale expression profiling, combining quantitative accuracy, with straightforward sample processing and control over culture conditions [17]. Not surprisingly, a wealth of information has been gained by investigating how several mammalian viruses manipulate their host's proteome, including adenovirus [18], influenza virus [19] and hepatitis C virus [20], among others. However, a large–scale quantitative proteomic analysis during BV infection has not been tackled to date.

In the present work, we investigate proteome expression changes in *Sf*9 cells associated with culture growth and BV infection. Since most of the host proteome is down–regulated at later times in the *Ac*MNPV life cycle, we focused on the transition from early to late phase (around 6 hpi) to investigate the establishment of infection. The lack of a complete genome sequence was overcome by cross–referencing to a database constructed from *S. frugiperda* and related insect species. For quantitation, cells were cultured in the presence of heavy arginine and lysine, commonly used SILAC reagents [17]. The adoption of biotechnologically relevant culture conditions in rich medium led to sub-optimal SILAC labelling, from which protein expression ratios were corrected by factoring the experimentally measured isotope incorporation in each protein. A statistical procedure is further described to handle data variability, revealing several proteins regulated by growth and infection that are hereby discussed. Our results provide a data repository that can help the scientific community to accelerate virological research of baculovirus infection and optimization of *S. frugiperda*-based bioprocesses.

## Results

### A draft *Sf*9 proteome

**Figure 1:**
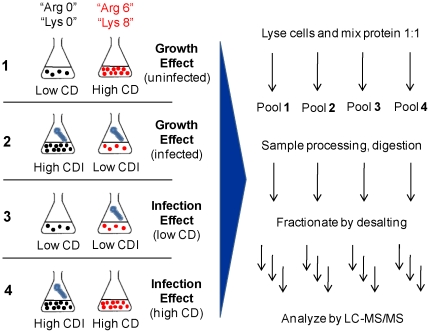
Schematic representation of the SILAC experimental design. For each comparison, cells maintained in unlabeled medium and medium labeled with heavy Arg (“Arg 6”) and heavy Lys (“Lys 8”) were grown to the desired cell density (CD) and infected (CDI) when appropriate. Protein extracts were mixed in equal amounts and subject to further processing until LC-MS/MS analysis.

**Figure 2:**
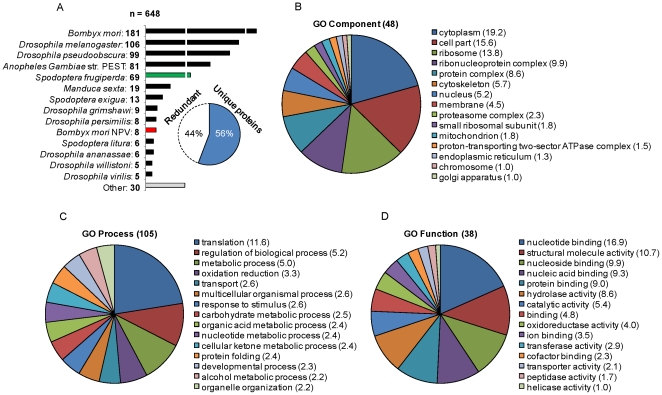
Species distribution and classification of the *Sf*9 proteome. (*A*) A total of 648 protein hits were retrieved from an insect subset of the NCBI non–redundant database. Due to high interspecies homology, some hits correspond to the same protein identified from different organisms. An estimated 56% correspond to unique PIRSF designations, indicating approximately 361 different *Sf*9 proteins. (*B*) (*C*) (*D*) Proteome classification according to the gene ontology (GO) vocabulary. A total of 48 Component, 105 Process and 38 Function terms were manually retrieved from the online resource *i*ProClass. The 15 most abundant terms in each class are shown. As each protein is generally assigned to more than one term, the percentage of proteins in each term is shown instead of total number to avoid redundancy.

The major challenge for a large-scale assessment of the *Sf*9 proteome is the lack of an annotated genome sequence. This missing knowledge was overcome by compiling a search database with available annotations from related insect species, constructed as a subset of the non–redundant protein database maintained by the National Center for Biotechnology Information (NCBI). The proteome of *Sf*9 cells was examined and its coverage was further maximized by enriching raw datasets with MS spectra recorded from different experimental conditions, biological replicates and biochemical fractionation of samples (Figure 1). When analyzing these MS spectra, a total of 648 high-confidence hits were identified as protein homologs from 31 species and 2 BV genomes (Figure 2A). In this database, protein sequence redundancy is defined such that the same *Sf*9 protein may be identified multiple times in closely related insect organisms. Thus, to provide an estimation of interspecies redundancy, the Protein Information Resource SuperFamily (PIRSF) classification system was adopted to group proteins sharing homology (common ancestry) and homeomorphy (full–length sequence and domain architecture similarity) [21]. 233 out of 418 proteins could be assigned to unique PIRSF designations, indicating a total of 56% different *Sf*9 proteins (approximately 361 out of 648).

Among the organisms with the highest number of identifications are the only sequenced lepidopteran, the silkworm *Bombyx mori* [22], and the model insects *Drosophila melanogaster* and *Anopheles gambiae*. Genomic information on *S. frugiperda* derives primarily from constructed EST libraries of larval hemocyte, fat body and midgut tissues, as well as the *Sf*9 cell line [23], [24]. As for viral genes, we identified 8 known early/late proteins from the related *Bm*NPV, which shares an average 90% homology with *Ac*MNPV [25]: P143 (helicase), DBP (DNA-binding protein) ME53, LEF3 (late expression factor 3), P35, HE65, 39K and GP64. In addition, 3 hits from the *Spodoptera litura* NPV correspond to the same P35 and the large (RR1) and small (RR2b) subunits of ribonucleotide reductase, a virally encoded enzyme involved in synthesizing deoxyribonucleotides [26]. However, the latter two genes are not present in the *Ac*MNPV genome, and the pronounced homology with their eukaryotic counterparts indicates that they originate from the cellular proteome [26], [27].

Figure 2B, 2C and 2D represent the proteome classification according to the gene ontology (GO) vocabulary. Since not all 648 entries could be assigned a PIRSF designation, the more general subset of proteins that could retrieve GO annotations using the online resource iProClass [28] was used. For an unbiased compilation, the 15 most abundant terms in each class are shown without further curation. It is shown that 576 proteins were assigned to at least one molecular function, 408 to at least one cellular component and 497 to one biological process, while 368 belong to the three categories. On the whole, the assigned categories are dominated by components of the translation machinery (ribosomal proteins, translation factors), followed by metabolic enzymes. Noteworthy, the proteome distribution across molecular functions and cellular components is substantially more heterogeneous than in the GO Process category, highlighting the higher degree of hierarchization in the former classes. Also, the number of different biological processes retrieved is much higher, mirroring the total number of terms available to date in each class. This indicates that the proteome here presented, though certainly incomplete, achieves a certain level of comprehensiveness.

Unfortunately, an enrichment analysis of GO annotations across the different database species is hindered by incomplete sequence information. For example, it is assumed that the *S. frugiperda* EST libraries contain at least 35% of the potential total gene number [24]. Moreover, the coverage of annotation is still poor, mostly focusing on high abundance proteins such as ribosomal proteins [23]. In our study, 51 of the 69 identified proteins directly assigned to *S. frugiperda* are ribosomal. The complete set of proteins with NCBI identifiers, species, PIRSF and GO assignments is provided as Table S1.

### Stable isotope labeling and quantitative analysis of protein expression ratios

*Sf*9 cells were cultured for at least 6 passages in customized medium where Arg and Lys have been replaced by their respective heavy isotope counterparts (^13^C)–Arg (“Arg 6”) and (^13^C^15^N)-Lys (“Lys 8”). In parallel, cultures in light media were maintained as a control. The feasibility of performing SILAC in this cell line was confirmed by comparable average duplication times (22–24 h), high viability (>90%) and normal morphology. To assess the effect of culture growth and *Ac*MNPV infection on the cellular proteome, a full factorial design of 2×2 comparisons was applied, whereby cells were grown to and infected at low (1.5−2×10^6^ cell/mL; LCD) and high (4−4.5×10^6^ cell/mL; HCD) cell densities (Figure 1). In infection experiments, cells were harvested at 6 h after virus inoculation. Synchronous infection using a high multiplicity of infection (MOI) is shown for LCD in Figure S1.

**Figure 3:**
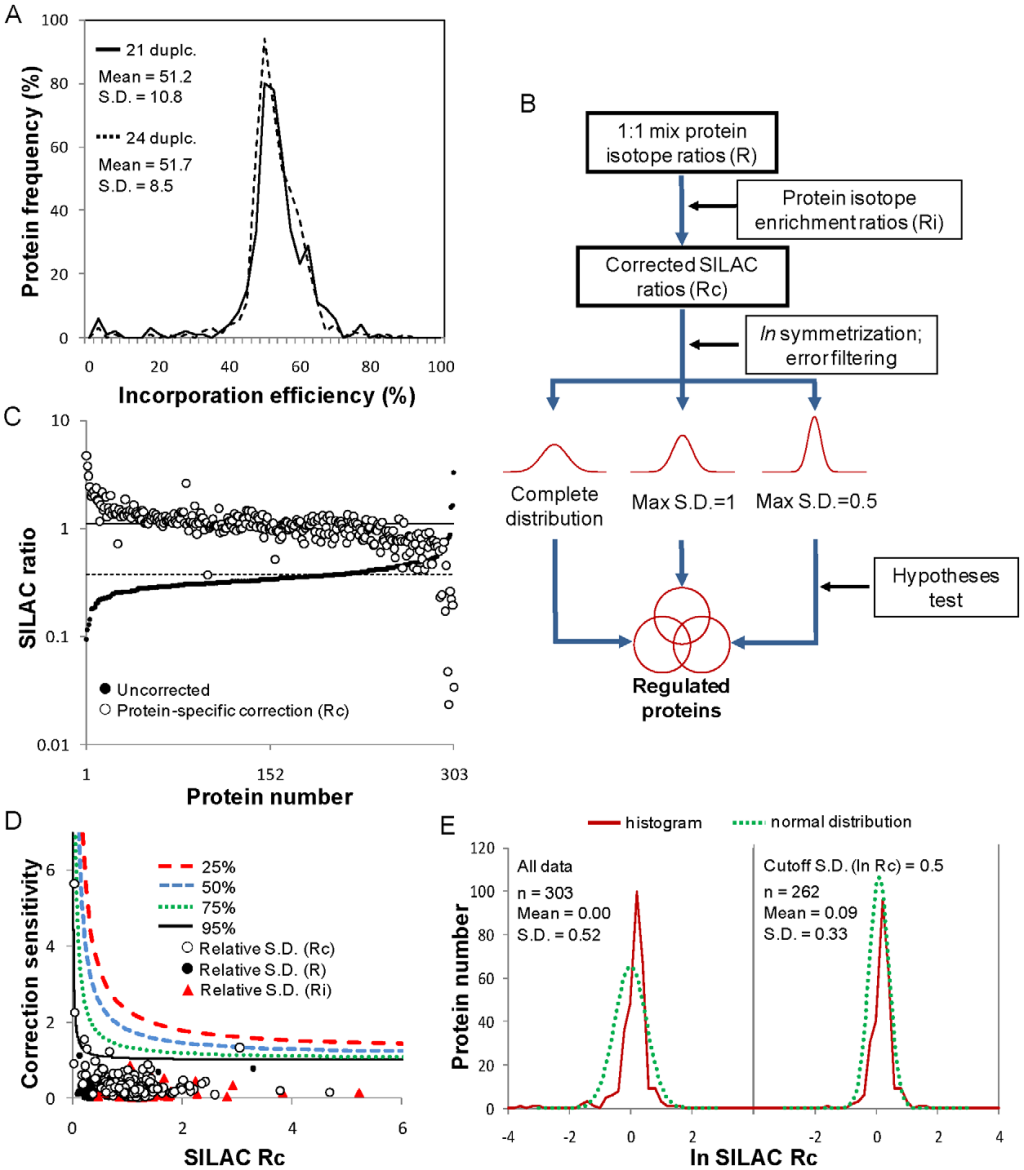
Correction and statistical analysis of SILAC ratios based on suboptimal label incorporation. (*A*) The proteome enrichment with heavy Arg and Lys was monitored after 21 and 24 cell duplications by analyzing unmixed protein extracts from labeled cultures. (*B*) Flow chart of the data analysis methodology. Briefly, protein isotope ratios directly obtained from LC-MS/MS analysis of 1∶1 mixed labeled/unlabeled cultures (R) are corrected in a protein-specific manner with the respective isotope enrichment ratios (Ri). The resulting set of corrected SILAC ratios (Rc) reflecting differential expression within each experimental comparison is symmetrized by logarithmic transformation and fitted with a Gaussian curve. In parallel, the unbiased standard deviations (S.D.) from triplicate experiments of R and Ri are propagated into standard deviations of Rc. Data distributions are then constructed by filtering out ratios with S.D. (*ln* Rc) values larger than 1 or 0.5, yielding more focused Gaussian fittings. For each distribution, differentially expressed proteins are defined as being different than the average by a *t*-test with at least 95% confidence. The combined set of proteins arising from the 3 distributions is considered to be regulated in the particular experimental comparison. (*C*) Protein-specific and global correction of ratios obtained from uninfected and infected cultures at low cell density. (*D*) The introduction of data variability by the mathematical operation was investigated by performing a sensitivity analysis of Rc. S.D. propagation shows a correlated trend of error amplification from R and Ri into Rc. (*E*) Added variability in the final *ln*–transformed distribution of corrected ratios is substantially reduced after applying a 0.5 maximum S.D. cutoff. Equations and definitions are available in Materials and methods.

A preliminary analysis of expected mass shifts of “Arg 6” and “Lys 8” clearly revealed the presence of unlabelled peptides. This was further confirmed by monitoring isotope incorporation into the proteome of the unmixed protein extracts Figure 3A). Incorporation efficiency was, on average, slightly over 50% after 21 cell duplications, and did not improve by an additional passage, suggesting that equilibrium was reached. We also performed cultures in media lacking Arg, Lys or both amino acids, and observed that the cells maintained high viability and the growth rates achieved in all media were comparable to those obtained when using medium containing both amino acids at 1.35 mM (Figure S2). However, there is a possibility that the culture medium contains undefined amino acid sources derived from plant or yeast hydrolysates, which are essential for cell growth and infection efficiency. In view of this, the primary heavy–to–light isotope ratios (R) were mathematically corrected with the measured ratios of incorporated label (Ri), adapting the method described in Liao et al. [29]. With this procedure (outlined in Figure 3B), the distribution of the resulting set of corrected SILAC ratios (Rc) was centered around 1 when uninfected and infected cultures at LCD were compared (Figure 3C). Deviations of individual proteins are more likely to reflect biological variability than different turnover rates, given the high number of cell duplications. To take this into account, SILAC ratios were corrected in a protein–specific manner, covering between 68% and 75% of the proteins quantified in each of the 4 comparisons. The remaining proteins were excluded to preserve quantitative accuracy.

Since all cultures were performed in triplicates, for each average R and Ri an associated standard deviation (S.D.) was calculated for most of the proteins. Naturally, each of these ratios constitutes a source of experimental error and biological variability in the calculation of Rc. The question therefore is whether the previously applied mathematical correction of the SILAC ratios introduces additional data variability by means of error amplification. It could lead one to assume that a statistical outlier has biological significance, or produce a distribution of corrected ratios that is too spread out, hampering the identification of differentially expressed proteins. To investigate this, a sensitivity analysis of Rc with respect to R and Ri was performed (Figure 3D). It can be seen that the lower the efficiency of label incorporation is, the more the error amplifies in the corrected ratios, increasing asymptotically for very low R, while being negligible for very high values. This heterogeneity is given by the non-linearity of the correction and correlates well with the experimentally propagated S.D. values associated with Rc. As hypothesized, the distribution of *ln*–transformed ratios after correction in the uninfected/infected comparison at LCD is too outspread (Figure 3E, left panel). In order to obtain a more focused normal fitting, the original data distribution was successfully decomposed by considering only proteins with S.D. (*ln* Rc) values lower than 1 or 0.5 (Figure 3B and 3E, right panel). For the proteins that lacked an experimental S.D. in either R, Ri or both, an estimation was calculated considering the average relative S.D. of the remaining proteins. This error filtering methodology allowed a considerable reduction in data variability, with comparable results obtained for the other 3 experimental comparisons.

For each transformed SILAC ratio distribution, a protein was considered to be differentially expressed when *ln* Rc was different from the average by a *t*-test with at least 95% confidence. This definition accounts simultaneously for the overall dispersion of the data as well as for the propagated S.D. associated with each corrected protein ratio, thus avoiding arbitrary definitions based only on location [18], [19], [20], [29]. For each protein, a different number of replicates (*n*) exists for uncorrected and incorporation ratios; those proteins that pass the test for the higher *n* value, but not for the lower one, were considered differentially expressed only if they lie outside of the bulk 95% of the data (Average ± 1.96 S.D.). This was also done for those proteins lacking a replicate for either R, Ri or both. Only protein ratios lying outside 68% of the distribution were considered to have biological relevance (Average ± S.D.). The combined set of proteins with statistically significant expression changes from the unfiltered and filtered distributions is considered to be the regulated sub-proteome for each experimental comparison (Figure 3B).

### Global assessment of growth- and infection-regulated proteins

As previously stated, two experimentally different sources of information regarding the impact of growth and infection on the cellular proteome are available. Yet, infection and growth constitute physiological “treatments” with substantially different strengths (comparing for instance the viral-induced halt in cell division with the cell density-associated decrease of growth rate). In the presence of experimental error and biological variability, their interacting effect can significantly obscure the assessment of how growth affects the cell proteome when comparing two infected cultures at different cell densities, for which reason we considered only uninfected cells in this case. Direct comparison of two infected cultures at different cell densities was carried out to discriminate possible changes in the expression levels of early BV proteins. On the other hand, the infection effect was evaluated not just separately for LCD and HCD cultures, but also globally by pooling together as “infection replicates” the six experimental comparisons performed in both conditions. Those proteins identified as differentially expressed from this pool will form a subset that has higher associated confidence.

According to the statistical analysis described, a total of 13, 14, 14 and 9 proteins had significantly changed expression along culture growth, infection (globally), infection at LCD and infection at HCD, respectively (Table 1, S2, S3, S4 and S5). Not surprisingly, some proteins only emerged as differentially expressed after applying S.D. filters to the distributions, hidden by the high variability in the original data sets (Figure S3). In addition, the only viral protein with significantly changed expression levels detected in this study was the late expression factor LEF3, found to be more expressed following infection at LCD than at HCD (Tables 1 and S2).

**Table 1:** List of proteins differentially expressed along culture growth and after BV infection.

Protein	GI number	Low CD/High CD	Inf./Uninf. (globally)	Uninf./Inf. (Low CD)	Inf./Uninf. (High CD)
**PDH-E3**	6014978	0.61±0.12 (5; 15.90%)		0.72±0.10 (3; 9.26%)	
**ALDH**	158291795		153.86±23.35 (1; 2.66%)		153.86±23.35 (1; 2.66%)
**ALDH**	195051749	0.17±0.08 (1; 2.51%)	2.85±1.07 (1; 2.51%)		
**PGDH**	158291584	17.75±3.67 (1; 4.98%)			
**ME**	153792270	20.80±4.74 (1; 4.25%)			
**SAHCH**	153791817	2.03±0.32 (2; 7.67%)	3.32±3.50 (2; 7.67%)		2.13±0.24 (2; 7.67%)
**SAHCH**	58381447			0.47±0.16 (1; 3.24%)	
**GP**	224999285		0.09±0.14 (5; 7.13%)		0.09±0.14 (5; 7.13%)
**HSDH**	114051868	3.08±0.69 (2; 7.88%)			
**eIF6**	114052170		0.56±0.08 (5; 29.39%)	1.79±0.25 (3; 16.73%)	
**eIF3k**	114053117		0.21±0.03 (1; 8.26%)	4.69±0.67 (1; 8.26%)	
**eIF3d**	112983254		0.25±0.14 (1; 2.19%)		0.25±0.14 (1; 2.19%)
**EF1b**	112982743			0.52±0.14 (2; 13.96%)	
**RPL4**	268306392		1.95±0.48 (3; 6.83%)	0.37±0.04 (3; 6.83%)	
**RPL19e**	125807113	0.31±0.03 (1; 9.36%)			0.33±0.03 (1; 9.36%)
**RPL23**	112984274	1.91±0.28 (3; 35.71%)			
**RPL26**	15213774	1.86±0.27 (5; 41.22%)			
**RPL24**	52783262		298.06±75.72 (1; 7.74%)		298.06±75.72 (1; 7.74%)
**RPS14b**	58383567			1.63±0.23 (2; 23.68%)	
**RPS3Ae**	126002490			2.15±0.40 (2; 9.33%)	
**RPS3**	16566719				0.52±0.11 (1; 6.17%)
**sHSP**	301070150		3.14±1.79 (1; 9.09%)	0.17±0.10 (1; 9.09%)	
**HSP70-5**	125981509	0.48±0.07 (5; 10.37%)			
**HSC70**	112984012			0.60±0.13 (2; 4.10%)	
**ERp57**	112983366		0.48±0.26 (2; 5.91%)		
**SRP54**	125980516		3.12±0.62 (1; 2.95%)		
**GTPase**	58381374	10.71±2.24 (1; 3.97%)			
**PP1c**	125773553	3.57±0.80 (1; 6.95%)			
**TNFR-AP1**	125811648	0.74±0.11 (1; 2.33%)			
**Mod-he**	163838692		0.60±0.15 (4; 21.80%)	1.82±0.22 (1; 4.94%)	
**Khc**	17136240			2.60±0.24 (2; 2.87%)	
**CLP**	21355917		10.87±0.93 (2; 16.49%)		21.40±3.29 (1; 7.98%)
**TRXL**	6560635			2.21±0.50 (1; 13.21%)	
**H2A**	24585673				0.36±0.15 (1; 8.87%)
LEF3^*^	9630874	0.57±0.11 (6; 21.30%)			

Average ratios correspond to corrected SILAC ratios (Rc) and are shown for under- or over–expressed proteins with statistical significance as described in the text. The number of peptide hits used for protein identification and overall protein coverage are shown in parenthesis for each comparison. CD - cell density. Un(inf) - un(infected). Protein name abbreviations: ALDH (aldehyde dehydrogenase), CLP (calponin–like protein), EF1b (elongation factor 1b), eIF(6; 3k; 3d) (eukaryotic translation initiation factor 6; 3k; 3d), ERp57 (endoplasmic reticulum protein 57), GP (glycogen phosphorylase), GTPase (guanosine triphosphatase), H2A (histone 2A), HSC70 (70 Kda heat shock cognate protein), HSDH (hydroxysteroid dehydrogenase), HSP70-5 (70 Kda heat shock protein 5), Khc (kinesin heavy chain), LEF3 (late expression factor 3), ME (malic enzyme), Mod-he (Mod(Mdg4)–heS0053), PDH-E3 (pyruvate dehydrogenase complex, E3), PGDH (6–phosphogluconate dehydrogenase), PP1c (protein phosphatase 1c), RPL(4; 19e; 23; 26; 24) (large subunit ribosomal protein 4; 19e; 23; 26; 24), RPS(14b; 3Ae; 3) (small subunit ribosomal protein 14b; 3Ae; 3), SAHCH (S-adenosyl-L-homocysteine hydrolase), sHSP (small heat shock protein), SRP54 (signal recognition particle 54), TNFR-AP1 (tumor necrosis factor receptor associated protein 1), TRXL (thioredoxin–like protein). *viral protein detected in the comparison of infected cultures at low and high CD.

**Figure 4:**
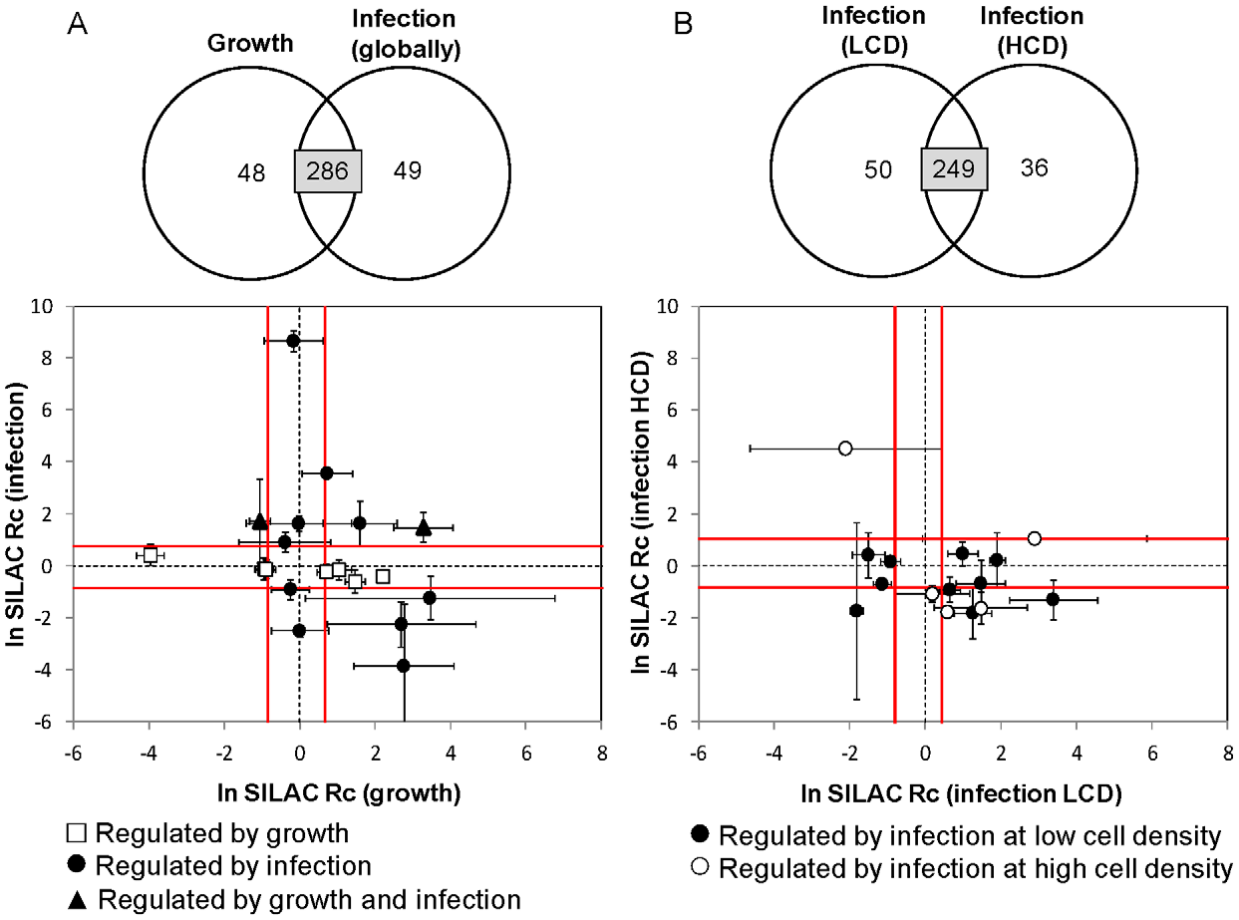
Comparative analysis of simultaneously quantified protein in the various experimental settings. Standardization of the *ln*-transformed corrected ratios was performed against the respective unfiltered distributions. Error bars presented for differentially expressed proteins correspond to propagated S.D. values from triplicate experiments. The vicinity of the average distributions is delimited by Average ± S.D. of the more focused distribution in each case (cutoff S.D. (*ln* Rc)  = 0.5), after standardizing as above. (*A*) (*B*) Comparative analyses of 286 and 249 proteins simultaneously quantified for the treatment pairs Growth/Infection (globally) and Infection (LCD)/Infection (HCD), respectively. Proteins considered to be regulated only by one condition are highlighted so they can be easily tracked in the other condition.

We also carried out a comparative analysis of the overall quantification results in the various experimental settings. The subsets of corrected SILAC ratios simultaneously quantified for the “treatment” pairs Growth/Infection (globally) and Infection (LCD)/Infection (HCD) were standardized and plotted against each other (Figure 4A and 4B). It is clear that proteins considered to be regulated only by one condition either have large S.D. values or locate close to the average, hence suggesting they were regulated in only one case. This is particularly interesting when infections at LCD and HCD are contrasted, since the absence of statistical significance in one of the conditions that is not due to high associated S.D. suggests an underlying cell density effect on BV infection. This phenomenon is well described as a reduced viral replication and overall bioprocess productivity at HCD compared with LCD infections, and was previously analyzed by the authors in terms of global metabolic flux patterns [30], [31], [32]. Nevertheless, viral infection is not expected to have an opposed effect on the expression of any given protein at different cell densities. This is confirmed by our data since when a protein is less expressed at LCD, it is not significantly over expressed at HCD, and *vice versa*.

### Regulatory modulations associated with culture growth and early baculovirus infection

**Figure 5:**
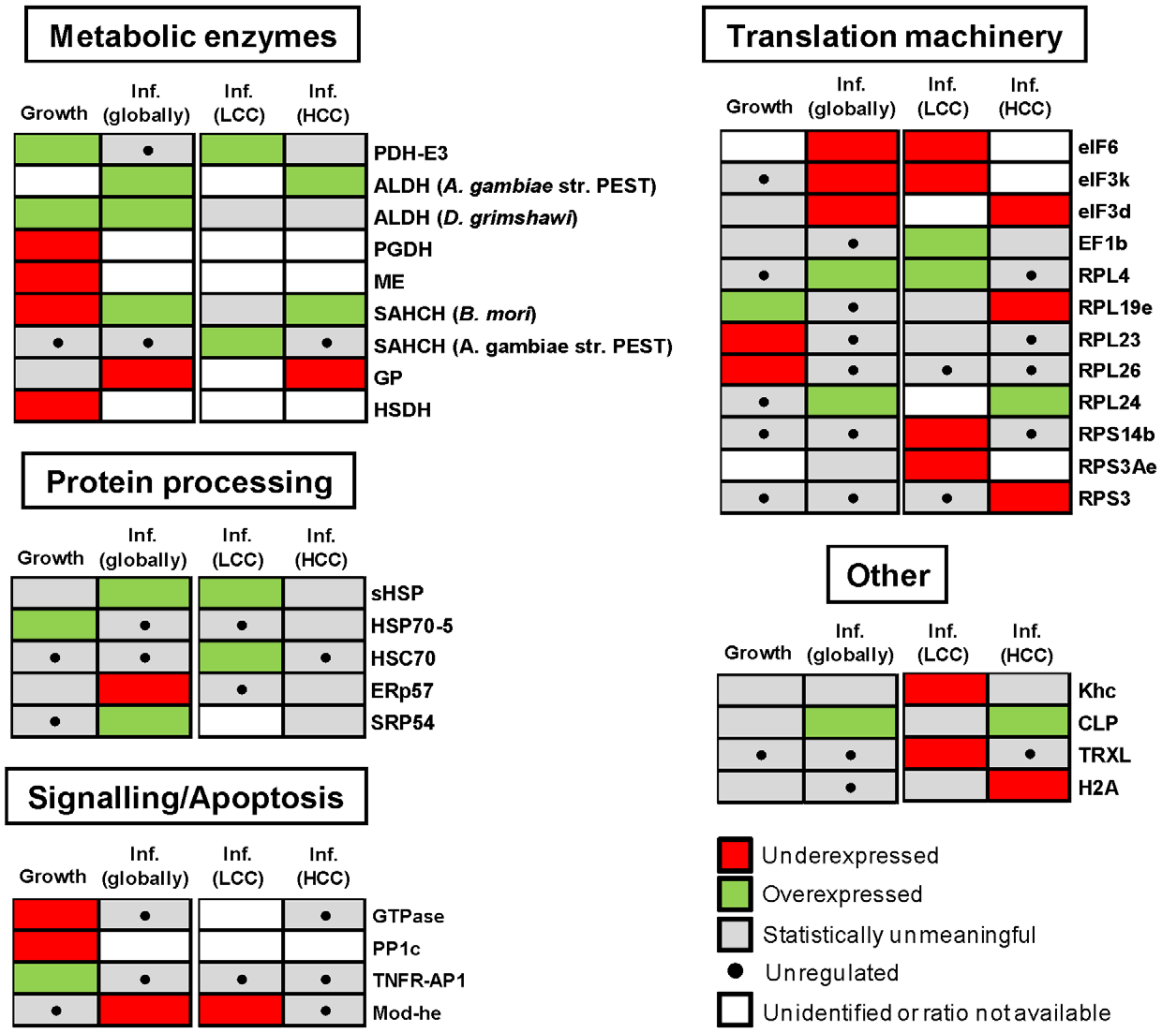
Functional organization of cellular proteins differentially expressed along culture growth and after BV infection. Under- or over-expression imply statistical significance as described in the text. Proteins suggested to be unregulated locate within Average ± S.D of the respective distributions (cutoff S.D. (*ln* Rc)  =  0.5). Expression ratios, number of peptide hits and protein coverage are shown in Table 1. For detailed statistical information see Tables S2, S3, S4 and S5.

Cellular proteins with changed expression levels across the 4 experimental comparisons were grouped according to their potential physiological roles in growth and infection (Figure 5). In conformity with the statistical analysis described, evaluating the relative strength of regulation among different proteins based directly on the corrected SILAC ratios could be misleading, due to the associated S.D. values. Thus, a simple code indicating up- or down-regulation was chosen to reflect the directional change.

Of the metabolic enzymes suggested to be regulated during culture growth, dihydrolipoyl dehydrogenase (E3 subunit of the mitochondrial PDH complex) and aldehyde dehydrogenase (ALDH, conversion of alcohols to carboxylic acids) are known to fuel energetic metabolism and were overexpressed at high cell densities. In contrast, several enzymes involved in different metabolic pathways were less expressed at HCD (6–phosphogluconate dehydrogenase – PGDH, malic enzyme - ME, S–adenosyl–L–homocysteine hydrolase – SAHCH and hydroxysteroid dehydrogenase - HSDH), consistent with the typical slowdown of growth along culture time. In particular, decreased anabolic fluxes through ME were previously reported by the authors [30]. Also relevant was the increased expression of the 70-KDa heat shock protein 5 (HSP70-5 or BiP), a resident ER factor that is up–regulated during the unfolded protein response (UPR) triggered by various cell stresses. Other changes associated with signaling pathways include down-regulation of a GTPase, the catalytic subunit of protein phosphatase 1 (PP1c) and the increased expression of tumor necrosis factor receptor associated protein 1 (TNFR–AP1), the later being involved in the transmission of apoptotic signals. Changes were also observed in some ribosomal proteins.

A total of 11 cellular proteins were differentially expressed at the same time for the global infection assessment and either of the LCD or HCD infection conditions, showing consistent regulation in all cases. This indicates that gross quantitative errors are not present in our study. In terms of metabolic enzymes, PDH-E3 and ALDH were upregulated in at least one infection condition, underlying the importance of the energetic metabolism previously described for BV replication [30], [32]. In opposition to culture growth, SAHCH was overexpressed after infection, an enzyme involved in methionine degradation and possibly associated with increased viral replication [33]. An interesting observation was the down–regulation of glycogen phosphorylase (GP) upon viral infection, an enzyme responsible for glycogen degradation and thus potentially important as an additional energy source. However, the apparent contradiction is in agreement with the fact that GP is repressed by dephosphorylation through PI3K–Akt signaling, a pathway stimulated very early in infection and shown to be essential for replication of BVs and other DNA viruses [34], [35]. This suggests a compromise between positive and negative outcomes from viral manipulation of functionally branched cellular pathways.

In terms of protein processing pathways, the up-regulation of HSP chaperones (sHSP, HSC70) has been associated with several viral infections, including BV [14], [15], most likely as a result of UPR initiation. Strikingly, our data indicates that central UPR effectors HSP70-5 and ERp57 are not over-expressed after infection, the later being even repressed, suggesting a previously unknown active role of BV genes in the modulation of ER stress. This is consistent with the observed up–regulated signal recognition particle SRP54, involved in targeting newly synthesized polypeptides for processing in the ER. The protein Mod(Mdg4) –heS00531, under expressed in this study, is part of the gypsy chromatin insulator complex and can trigger apoptosis by a specific pathway known to be blocked by BV IAP and P35 proteins [36]. Other interesting discoveries concern the modulation of proteins involved in cytoskeleton motility, underscoring its importance for the BV life cycle, and the down-regulation of a thioredoxin-like protein (TRXL) involved in oxidative stress, among histone H2B and several translation initiation factors.

Finally, of the proteins suggested to be only regulated by infection at LCD, the physiological roles of HSC70, Mod(Mdg4)–heS00531 and TRXL may imply limitations to BV replication during high cell density culture. In particular, the lower levels of LEF3 detected following infection at HCD, compared to cells infected at LCD, are consistent with the lower baculovirus progeny produced after infection at HCC when compared with LCC [30]–[32]: LEF3 is one of 6 virally encoded genes (called replicative LEFs) that are essential for DNA replication [1].

## Discussion

In this work, a comprehensive SILAC-based proteome analysis of the popular *Sf*9 cell factory was presented. The effect of BV infection and culture growth on the cellular proteome was investigated to gain insights into regulatory modulations associated with a productive infection cycle, thus relevant from both the virological and bioprocess points of view. Despite not having a complete genome sequence for this insect species, homology-based mass spectra interpretation gave the framework for genome–wide data extraction from limited knowledge. Inarguably, reanalyzing the data generated against a complete *S. frugiperda* database would unravel many more important players in this complex molecular interaction. To this respect, we expect this work to constitute a potential catalyst for further developments in the BV-*Sf*9 system.

The analysis of differentially expressed proteins in SILAC experiments is straightforward when an almost complete incorporation (>95%) of heavy amino acids in cellular proteins is possible. However, atypical growth behavior of certain cellular types may complicate or completely prevent this ideal scenario [29], [37]. Compared to mammalian cells, the metabolic capacity for amino acids biosynthesis in insect cell lines is less known, with reports pointing to a greater flexibility [38], [39]. Our results are not conclusive on this point given the presence of undefined amino acids sources in serum-free culture medium. Alternative chemically defined media for growth of *S. frugiperda* cell lines require supplementation with serum or yeastolate. Cell adaptation to a minimal supplementation of these components could be cumbersome and induce alterations to normal culture behavior, affecting the infection cycle. Furthermore, serum dialysis was recently shown to induce marked changes in the proteomes and phosphoproteomes of several mammalian cell lines [40]. Instead of invasive cell culture manipulation, the mathematical correction of the primary SILAC ratios with experimentally measured enrichment ratios can be profitably applied to analyze industrially relevant culture conditions, while preserving quantitative accuracy. Further, the statistical implications of this procedure were thoroughly analyzed and a process to circumvent added variability in the final data sets caused by error amplification was described, which would otherwise preclude the identification of several proteins with changed expression levels. This bears relevance for studies where for various reasons complete protein labeling is not attainable.

Our study was conducted at 6 hpi to assess cellular proteomic modulations associated with the establishment of infection, which will ultimately determine the outcome in terms of viral replication and recombinant protein expression. At this stage in the infection life cycle, Nobiron et al. [14] and Salem et al. [15] showed most host transcript levels are not pronouncedly changed, suggesting a post–translational regulation (an exception are HSP70 protein family members, such as HSC70, which are increased). Here we report protein expression changes in several cellular pathways. For example, three metabolic enzymes are shown to be up-regulated after infection, emphasizing the dependency of BV replication on cell metabolism. In particular, a higher TCA cycle activity driven by an efficient glycolytic bridge (PDH-E3) and anaplerotic feeding of carboxylic acids (ALDH) is important for efficient energy generation and diversion of substrates to lipid biosynthesis. This agrees with the metabolic flux analysis studies previously carried out by our group [30], showing increased fluxes through the main central carbon metabolism (glycolysis, TCA cycle), and is supported by the finding that increased BV productivities are obtained when carboxylic acids are supplemented to the culture along with infection [32]. Other viruses were also found to actively modulate energetic and anabolic pathways in proteomic studies. Of notice, Diamond et al. [41] have showed through trypsin–catalyzed ^18^O sample labeling that the hepatitis C virus significantly over expresses glycolytic and TCA cycle enzymes for energy generation, while also over expressing non–oxidative enzymes in the pentose phosphate pathway for biosynthesis. Furthermore, the same central catabolic pathways were observed to be activated following human cytomegalovirus infection in a metabolic flux profiling study, as well as the efflux to fatty acid biosynthesis necessary for viral envelope formation [42]. Collectively, these findings contribute to the view that instead of representing a simple metabolic burden, viruses actively regulate the metabolic pathways essential for their replication, therefore behaving as natural metabolic engineers [43].

The induction of energetic metabolism as a response to culture growth was also observed, a phenomenon previously described to be associated with a low proliferative, quiescent physiological state [44], and which represents an apparent paradox in relation with the mentioned negative cell density effect on BV replication. However, this can be explained by a concomitant growth–associated repression of anabolic processes (for instance PGDH and ME decreased in this study) and reduced substrate uptake rates previously reported [30], [31], consistent with an overall lower metabolic rate.

The early up-regulation of HSC70 and the related sHSP after LCD infection (but not HCD) indicate a disturbance of ER homeostasis known to be associated with viral infections. Interestingly, we observed expression changes in two proteins that together are counterintuitive with an activation of UPR, namely decreased levels of the chaperone ERp57, a central UPR effector also involved in Ca^2+^ homeostasis, and the polypeptide transporter SRP57. These findings suggest that, through unknown mechanisms, BVs are able to take advantage of ER stress while avoiding negative effects associated with UPR, probably in different ways than already described for other viruses [45], [46]. Failure to induce HSC70 expression at HCD infection can mean less ER capacity for viral protein processing and thus less BV progeny.

Also interesting are the decreased levels of TRXL (only after LCD infection), since human thioredoxin (TRX) related proteins are known to integrate signals and prevent apoptosis from ER, oxidative and mitochondrial stresses. This complexity draws further curiosity into the strategies that different viruses have evolved to manipulate the cell for their advantage. For instance, the endogenous human T-cell leukemia retrovirus type I was shown to promote cell growth through elevation of TRX levels, while the apoptotic HIV infection is associated with decreased TRX expression [47]. In contrast, Popham et al. [16] observed unaltered levels of TRXL in the lepidopteran *Heliothis virescens*, although at very late times after *Ac*MNPV infection. More relevant, however, is the discovery that the multifaceted early P35 protein also acts as an antioxidant by quenching ROS [48], reinforcing the fact that an induction of oxidative stress is only observed late in infection [49]. One hypothesis is that this additional role of P35 compensates for decreased TRXL levels in preventing oxidative stress–induced apoptosis early in infection. Alternatively, the possibility of a direct viral–mediated regulation of TRXL levels concurs with unaltered levels of this protein following HCD infection, when viral replication is less efficient. An involvement of P35 in the modulation of TRXL, as it seems conclusive in the case of Mod(Mdg4)–heS0053, remains to be elucidated.

The role of LEF3 as a key determinant of a productive infection cycle is suggested by its lower expression at HCC compared to LCC infections. Noteworthy, a recent study has demonstrated that silencing of the replicative LEFs (including LEF3) during AcMNPV infection not only blocks viral DNA replication and late gene expression, but also prevents the induction of apoptosis and shutoff of cellular protein synthesis in *S. frugiperda* cells [50]. Hence, this protein can be a potential target to overcome the negative cell density effect on baculovirus production. Moreover, one may speculate a link between energetic metabolism and the differential accumulation of other viral–encoded proteins in regulating the level of baculovirus replication and recombinant protein expression.

Finally, among changed expression levels in several translation factors and ribosomal proteins, we found diminished levels of kinesin heavy chain (Khc), a tubulin motor protein, and up-regulation of a calponin-like protein (CLP), an inhibitor of myosin ATPase activity, which in turn is an actin motor protein. These observations suggest BVs reorganize the cytoskeleton not only through actin polymerization [5], [51], but possibly also by regulating other proteins to enhance their own intracellular transport.

In conclusion, as a hypotheses generation tool, this proteomic study offers new insights and potential research targets for a better understanding of the complex BV infection cycle. One exciting point remains to be discriminating active modulations induced by the virus and the natural cellular response to infection. The complete resolution of this puzzle will probably require complementary information from targeted, scale-down experiments and other high-throughput assessments of protein-protein, protein-DNA and protein phosphorylation networks. From a distinct perspective, our data highlights possible regulatory bottlenecks associated with the described cell density effect on viral replication. This is one of few examples where large–scale quantitative proteomics was used with a focus on improved performance of an animal cell factory, in the scope of Systems Biotechnology. In a broader sense, this work provides a case–study of the application of advanced “omic” technologies in poorly characterized organisms.

## Materials and Methods

### Stable isotope labeling of *Sf*9 cells and experimental cultures

The commercially available *Sf*9 insect cell line (ECACC 89070101) was kindly provided by Dr. Otto W. Merten (Généthon, France). Culture maintenance was done as previously described [32]. For labeling, customized serum- and protein–free SF900II medium (Invitrogen, Life Technologies) without Arg and Lys was supplemented with (^13^C)-Arg and (^13^C^15^N)–Lys at 1.35 mM each (Cambridge Isotope Laboratories). Cells were cultured in this medium for 5 passages growing as 20 mL flask suspension cultures and then up-scaled to 70 mL for an additional 1–2 passages. In parallel, cells were cultured in light medium containing (^12^C)–Arg and (^12^C^14^N)–Lys at the same concentration. For infection experiments, cells were grown to the desired cell density and inoculated with a recombinant *Ac*MNPV at a MOI of 5 infectious particles per cell. Information on virus composition, handling and quantification are provided in Carinhas et al. [31]. About 100 million cells were harvested either before infection or at 6 h post–infection and seeded by centrifugation. Cell pellets were washed with ice–cold PBS, instantly frozen and stored at −85°C until analysis.

### Sample processing

Cells were lysed in basic RIPA buffer containing 25 mM Tris (pH 7.6), 150 mM NaCl, 1% NP-40, 1% Na deoxycholate, 1 mM EDTA, and supplemented with 1 mM PMSF and 1x complete protease inhibitor cocktail (Roche, Switzerland). Before further handling, the protein content was quantified for each extract (BCA Protein Assay Kit, Thermo Scientific, USA) and equal protein amounts of labeled and unlabeled material from each culture were pooled. Incorporation of iosotopically labeled amino acids was checked on pooled samples from light and heavy cultures as well as unmixed samples. For this, proteins from the lysate were precipitated by adding 9 volumes of ice–cold 13.3% trichloroacetic acid/0.07% β-mercaptoethanol (β-ME) in acetone and incubated overnight at −20°C. Protein content was collected by centrifugation, washed twice with 90% acetone/0.07% β-ME and dissolved in 50 mM Tris–HCL/8 M urea (pH 8). Samples were reduced with 10 mM dithiothreitol and alkylated with 50 mM iodoacetamide, followed by overnight dialysis against 50 mM Tris–HCL/6 M urea (pH 8). Before trypsin digestion, the dialysate was first digested with 20 µg of Lys-C at 30°C for 2 h and then diluted with 5 volumes of 50 mM Tris-HCl (pH 8). Trypsin digestion was carried out overnight at 37°C using a 50∶1 protein to enzyme ratio. The peptide mixtures were acidified and approximately 1 mg of the initial protein content was desalted in 0.1% trifluoroacetic acid (TFA) using MacroSpin Column, Silica C18, 30–300 ug capacity (The Nest Group, Inc., MA, USA). Peptides were biochemically stepwise eluted with 0.1% TFA containing 20%, 40%, and 60% acetonitrile. Before LC–MS/MS analysis, peptide fractions were dried in a speed vac and dissolved in 0.1% TFA.

### LC-MS/MS analysis, database construction, searching and isotope ratio quantification

LC–MS/MS analysis was performed on a LTQ-Orbitrap hybrid instrument (Thermo Scientific, San José, CA, USA). 2 µl of peptide digest were injected with an autosampler (CTC Analytics, Agilent Technologies) onto a C18 trapping column (ProteoCol C18 0.15×10 mm, SGE, Ringwood, AU) that was connected to a separation column (0.1 mm×10 cm) packed with Magic 300Å C18 reverse-phase material (5 µm particle size, Michrom Bioresources, Inc., Auburn, CA, USA). A linear 80-min gradient from 2 to 50% solvent B (80% acetonitrile/0.1% acetic acid) in solvent A (2% acetonitrile/0.1% acetic acid) was delivered with an Agilent 1200 nano pump (Agilent, Basel, Switzerland) at a flow rate of 300 nL/min. The eluting peptides were ionized at 1.7 kV. The LTQ-Orbitrap was operated in data-dependent mode. A survey scan between m/z 400–1600 was acquired in profile mode in the Orbitrap at 60,000 resolution and the 10 most abundant ions were then selected for fragmentation in the LTQ part of the instrument at normalized collision energy of 35%. Singly charged ions were omitted from fragmentation and previously selected ions were dynamically excluded for 25 sec. Scan-to-scan calibration was allowed by setting the lock mass to m*/*z 445.120025 [52].

The LC-MS/MS data were searched with the SEQUEST search engine (version 3.3.1 SP1) [53] against an indexed insect database constructed as a subset of the non–redundant protein database maintained by NCBI (October 1^st^, 2010): all protein sequences for the insect species *Manduca*, *Drosophila*, *Anopheles*, *Bombyx*, *Spodoptera*, *Adisura*, *Culex*, *Heliothis*, *Agrotis*, *Plubella*, *Apis* and *Mamestra* were extracted from the non-redundant database and compiled into an insect–specific database. To this, a second database was collated having all protein sequences whose annotations contained the text string “insect” as well as baculovirus protein sequences.

The precursor ion and fragment ion mass tolerances were set to 10 ppm and 0.6 Da, respectively. Two missed cleavages were allowed. Dynamic modification was set for oxidized methionine, while cysteine carbamidomethylation was set as a fixed modification. The results were filtered for Xcorr values of 1.5 for singly, 2.0 for doubly, 2.5 for triply, and 3.0 for quadruply charged peptides. Delta CN was set to 0.1, and the peptide and protein probabilities were set to 0.5 and 0.01, respectively. In addition, all single peptide hit identifications were inspected manually for the correctness of identification.

For SILAC ratio quantification, the area of those precursor ions that had been identified by SEQUEST were integrated by the PepQuan option of Bioworks (Thermo Scientific, version 3.3.1 SP1). Precursor ion and fragment ion mass tolerances were set to 10 ppm and 0.6 Da, respectively. Searched files corresponding to different fractions of the same sample were merged together. Relative abundances were calculated as area ratios of heavy and light peptides, and the protein ratios were calculated as averages of all quantified peptides. The mass shift tolerance of “Arg 6” and “Lys 8” was set to 0.02 Da.

### Data analysis

The corrected unlabeled/labeled culture ratio for each protein was derived from Liao et al. [29] as follows: 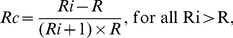
(1)


where R represents the heavy/light isotope ratio from a 1∶1 mixed sample and Ri the heavy/light isotope ratio from the corresponding unmixed labeled sample. The error propagation from R and Ri into Rc is based on the unbiased standard deviation from triplicate experiments and was calculated according to the equation:
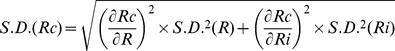
(2)


Sensitivity analysis of equation (1) was performed by calculating the relative gradient with respect to R and Ri:
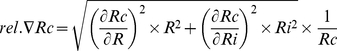
(3)


The corrected SILAC ratio distributions were symmetrized around 0 through logarithmic transformation. Using the natural logarithm (*ln*) has the advantage of preserving data dispersion by only dividing each S.D. (Rc) by the respective Rc to yield S.D. (*ln* Rc). Maximum S.D. (*ln* Rc) cutoffs were then applied to each data set resulting in more focused normal distributions. The two-tailed Student's *t*-test was performed assuming unequal variances and unequal sample sizes.

## Supporting Information

Figure S1 **Synchronous infection of** ***Sf*** **9 cells.** Cells were infected at 1.5−2×10^6^ cell/mL by adding 5 viral particles per cell (MOI 5). A high-titer virus stock was used to ensure minimal culture dilution. The percentage of GFP-positive cells was followed by flow cytometry, monitoring GFP expression under the control of the very late *polh* promoter. Synchronous infection can be observed by discounting 18–24 h from viral inoculation until full *polh* activity.
(PDF)

Figure S2 ***Sf*** **9 cells growth profile in customized SF900 II medium.** Cells were grown either without Arg, Lys or both amino acids. Medium supplemented with 1.35 mM Arg and 1.35 mM Lys was used as control. Undefined medium components may contain variable amounts of these amino acids.
(PDF)

Figure S3 **Allocation of differentially expressed proteins for each experimental comparison after the application of S.D. (** ***ln*** **Rc) cutoffs to data distributions.**
(PDF)

Table S1 Complete set of identified proteins with NCBI identifiers, species, PIRSF and GO assignments.
(XLS)

Table S2 Quantitative and statistical information on proteins with changed expression levels along culture growth.
(XLS)

Table S3 Quantitative and statistical information on proteins with changed expression levels as a response to infection (globally).
(XLS)

Table S4 Quantitative and statistical information on proteins with changed expression levels as a response to infection at low cell density.
(XLS)

Table S5 Quantitative and statistical information on proteins with changed expression levels as a response to infection at high cell density.
(XLS)
