# Supplementary figures and images for: Quantitative Proteomics of Spodoptera frugiperda Cells during Growth and Baculovirus Infection
Source: PLoS One. 2011 Oct 18;6(10):e26444. doi: 10.1371/journal.pone.0026444 (PMC3196586; doi:10.1371/journal.pone.0026444)

**Figure S1**

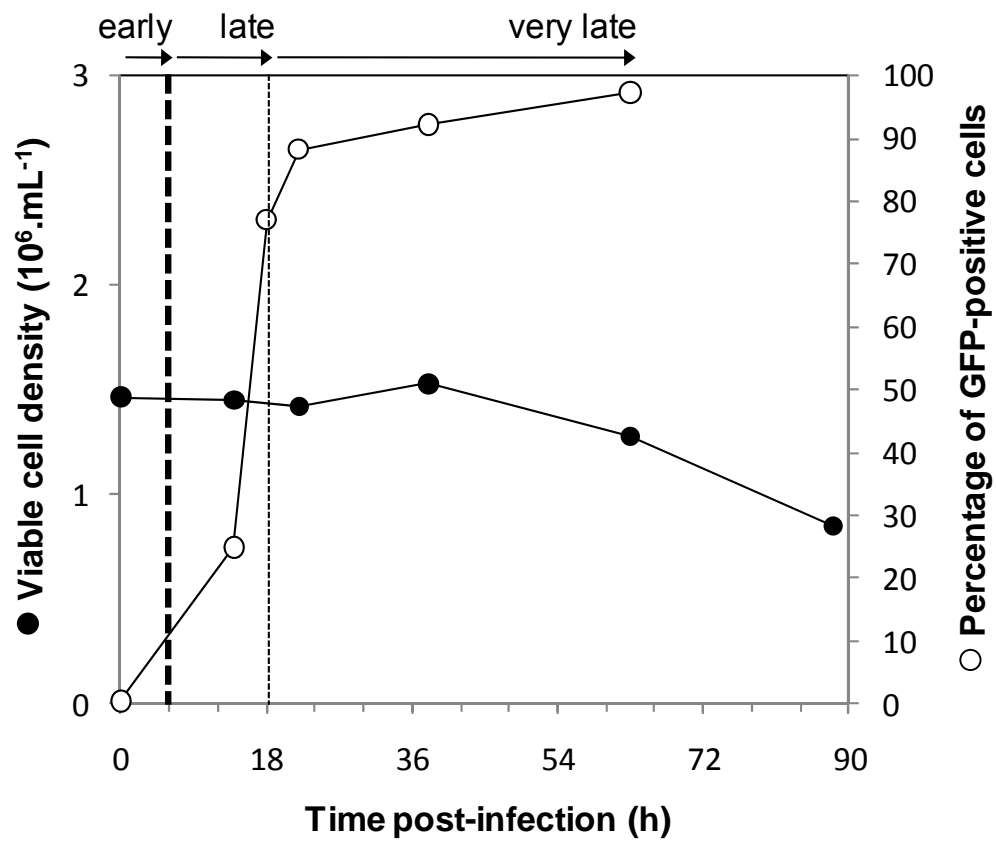

Supplement: Figure S1 — Synchronous infection of Sf 9 cells. Cells were infected at 1.5−2×10^6^ cell/mL by adding 5 viral particles per cell (MOI 5). A high-titer virus stock was used to ensure minimal culture dilution. The percentage of GFP-positive cells was followed by flow cytometry, monitoring GFP expression under the control of the very late polh promoter. Synchronous infection can be observed by discounting 18–24 h from viral inoculation until full polh activity. (PDF) [file pone.0026444.s001.pdf]

**Figure S2**

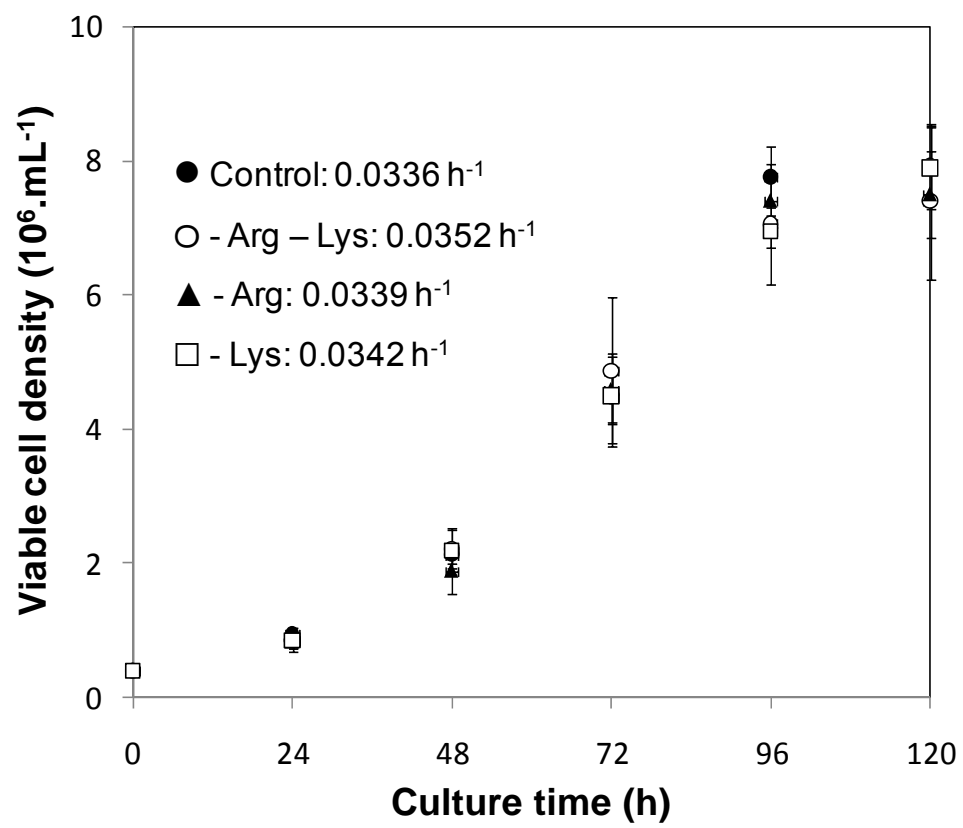

Supplement: Figure S2 — Sf 9 cells growth profile in customized SF900 II medium. Cells were grown either without Arg, Lys or both amino acids. Medium supplemented with 1.35 mM Arg and 1.35 mM Lys was used as control. Undefined medium components may contain variable amounts of these amino acids. (PDF) [file pone.0026444.s002.pdf]

Figure S3

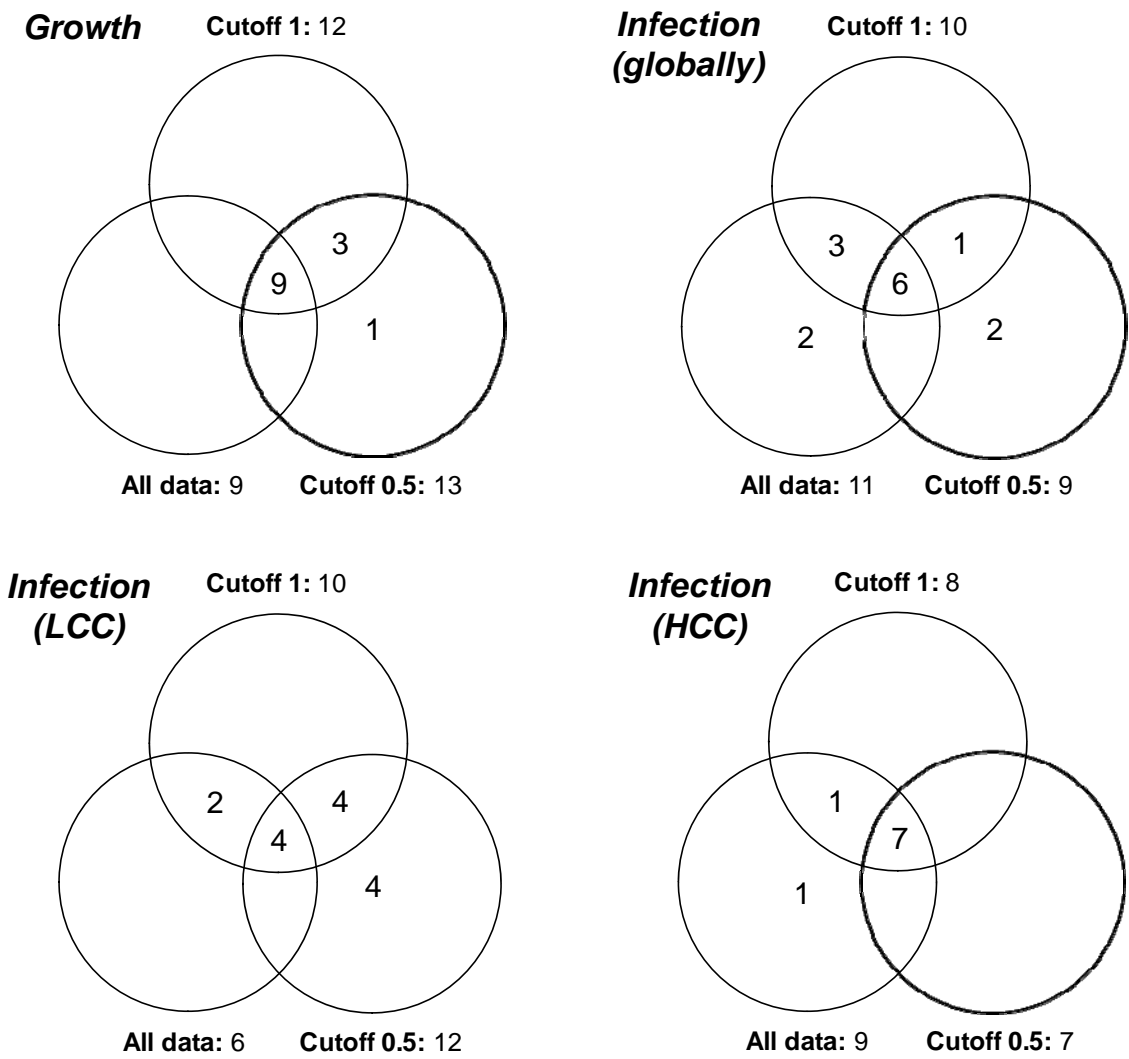

Supplement: Figure S3 — Allocation of differentially expressed proteins for each experimental comparison after the application of S.D. ( ln Rc) cutoffs to data distributions. (PDF) [file pone.0026444.s003.pdf]
